# Scientific investigation of a fractional model based on hybrid nanofluids with heat generation and porous medium: applications in the drilling process

**DOI:** 10.1038/s41598-022-10398-3

**Published:** 2022-04-20

**Authors:** Dolat Khan, Poom Kumam, Ilyas Khan, Arshad Khan, Wiboonsak Watthayu, Muhammad Arif

**Affiliations:** 1 Department of Mathematics, Faculty of Science, King Mongkut’s University of Technology Thonburi (KMUTT), 126 Pracha Uthit Rd., Bang Mod, Thung Khru, Bangkok, 10140 Thailand; 2 Center of Excellence in Theoretical and Computational Science (TaCS-CoE), Faculty of Science, King Mongkut’s University of Technology Thonburi (KMUTT), 126 Pracha Uthit Rd., Bang Mod, Thung Khru, Bangkok, 10140 Thailand; 3 Department of Medical Research, China Medical University Hospital, China Medical University, Taichung, 40402 Taiwan; 4 Department of Mathematics, College of Science Al-Zulfi, Majmaah University, Al-Majmaah, 11952 Saudi Arabia; 5 Institute of Computer Sciences and Information Technology, The University of Agriculture, Peshawar, Pakistan

**Keywords:** Nanoparticles, Nanoscience and technology, Fluid dynamics

## Abstract

This article investigates graphite-aluminum oxide hybrid nanoparticles in water-base fluid with the addition of heat generation in the presence of a porous medium. The problem is formulated in terms of momentum and energy equations with sufficient initial and boundary conditions. The solution is investigated by using the Laplace transform method. It is observed that the velocity of the drilling fluid is controlled by adding hybrid nanoparticles as compared to simple nanofluids. In a similar way, the temperature of the fluid is reduced. Also, the heat transfer rate is boosted up to 37.40741% by using hybrid nanofluid compared to regular nanofluid. Moreover, the heat transfer rate was increased up to 11.149% by using different shapes of nanoparticles in the base fluid water. It is also observed that by using hybrid nanofluid skin fraction is boosted up at y = 0 and boosted down at y = 1.

## Introduction

Heat transfer plays an important role in several manufacturing industries, hearing transition procedures. In the recent era of research, some researchers report the analysis of heat transfer in different fluids which is solved by exact analysis and numerical techniques^1–5^. In various heat transfer systems, fluids such as water, ethylene glycol, alcohol, and oils are known as heat carriers. But because of the low thermal conductivity, they are not a strong heat carrier for these fluids. The scientist's key task was to increase the thermal conductivity of the fluids^6^. One of the approaches normally used in practice to deal with this problem is to maximize the heat exchanger's contact area. However, this approach takes to an unnecessary rise and scientific futility of heat control in the system of heat transport. To control this situation, Choi^7^ was the first, who introduced the idea of nanofluids for the improvement of thermal conductivity. From this motivation, many researchers have involved nanofluids in their research to improve the thermal conductivity of fluids in daily life problems^8–12^. Among them, Sheremet et al.^13^ reported Aluminium oxide water nanofluids for the application of solar collectors, where the problem is solved by a numerical scheme named the finite difference method. Furthermore, Ali et al.^14^ find the exact analysis of nanofluids through the Laplace transform method. They used different types of nanoparticles i.e., Aluminium oxide, titanium oxide, copper oxide, etc. in rotating frames for the application of solar collectors, to improve the efficiency of solar collectors. Krishna and Chamkha^15^ used nanofluids for the application in biomedical engineering along with the Hall effects on magnetohydrodynamic (MHD) unsteady flow, which helps in the cancer treatment process. Molana^16,17^ added a comprehensive review on nanofluids as an application in heat exchangers and for the performance of thermal enhancement. The authors reported the information on the size and materials of nanoparticles, length, base fluid, flow regime, Reynolds number, and concentration used during research. Sajid and Ali^18^ reported a critical review of nanofluids, on the application of advanced and more recently used heat transfer devices. The results of different parameters on the improvement of thermal conductivity using nanofluids have been studied for the devices. Heat transfer equipment mentioned in this study includes a rotating tube, radiators, heat exchangers, tube heat exchangers, heat sinks, and shells. Various associations are often compiled, correlated, and analyzed, which are used for investigational verification or established in reviewed studies. Because normal fluids have low thermal conductivity as compared to nanofluids. As for the formation of ions, nanomaterials are used to improve the expertise in the heat transfer of normal fluids, which subsequently upsurges thermal conductivity. Since then, multiple progressions have been tracked by nanofluids, with various classes.

By preparing hybrid (composite) nanoparticles, the thermal conductivity of the nanoparticles can be altered or modified. Hybrid nanofluids are classified as base fluids that consist of two or more distinct nanometer-sized materials. A brief review of the preparation, heat transfer, friction factor, and thermal properties are reported by Sundar et al.^19^. Sidik et al.^20^ reported the method of planning, thermophysical properties, the efficiency of hybrid nanofluid heat transfer, and method of stability investigation in various applications of heat transfer. In addition, this report discusses hybrid nanofluid problems and several recommendations to enhance future studies in this area. Ghadikolaei et al.^21^ examined the mathematical analysis of hybrid nanofluid over a stretching sheet along with MHD. The combination of titanium dioxide and copper nanoparticles is taken along with water as base fluid. The solution is obtained via Runge–Kutta (R–K) Fehlberg method. Furthermore, in the recent era, many researchers have used different hybrid nanofluids with different combinations of nanoparticles for various applications. Recently, some of them are, Yildiz et al.^22^, Hayat and Nadeem^23^, Muhammad^24^, Khan et al.^25^, etc. Moreover, Khan et al.^26^ used clay nanoparticles along with heat transfer to the water cleaning process. The problem was solved by using the Laplace transform method. To investigate the combined effects of heat and mass transfer on the flow of water functionalized oxide and non-oxide nanofluids across porous media, as well as thermal radiation and chemical reaction effects in MHD, is reported by Hussanan et al.^27^. Authors used here three types of basic fluids: water, kerosene, and motor oil, all of which include various forms of oxide and non-oxide nanoparticles. Combining copper (Cu)–Aluminium oxide (Al2O3)/water hybrid nanofluid and magnetic field to investigate the thermal efficacy of half-sinusoidal nonuniform heating at different spatial frequencies for a porous natural convection system is reported by Biswas et al.^28^. Some other researchers also studied water-base hybrid nanofluid for different purposes in different fields^29–32^.

**Figure 1:**
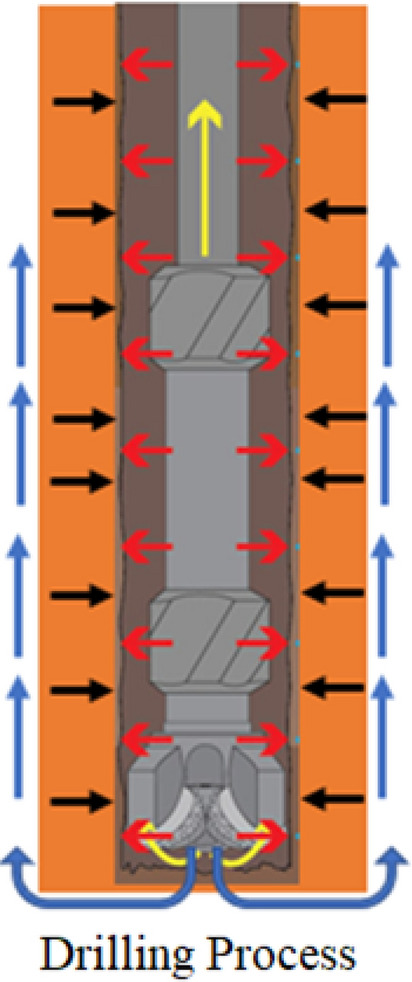
Schematic representation of the drilling process.

As mentioned above, it is very clear that hybrid nanofluid has numerous applications in different fields. Besides this motivation, the novelty of this paper is to examine the scientific investigation of fractional mathematical analysis of hybrid nanofluid as an application in the water filtration process. For this purpose, the combination of graphite and aluminium oxide nanoparticles with water-based fluid is considered in the existence of porous medium and heat generation. The exact solution is investigated by the Laplace transform method. For several reasons, the solutions obtained here are significant. These solutions can be used to verify the precision of their results by experimentalists in various water filtration process applications. Engineers can utilize these findings to decrease the number of experiments and time spent on practical work by altering different factors during the drilling process. $Al_{2} O_{3}$ was chosen for its capacity to improve drilling mud rheological, electrical, and thermal characteristics. Because of their shape, graphite ($Gr$) is used to offer reduced filtrate loss. Furthermore, the $Al_{2} O_{3} ,\,Gr$ combination is non-hazardous and ecologically safe, making it ideal for usage in sensitive settings and applications, the drilling process is represented in Fig. 1.

## Model formulation

**Figure 2:**
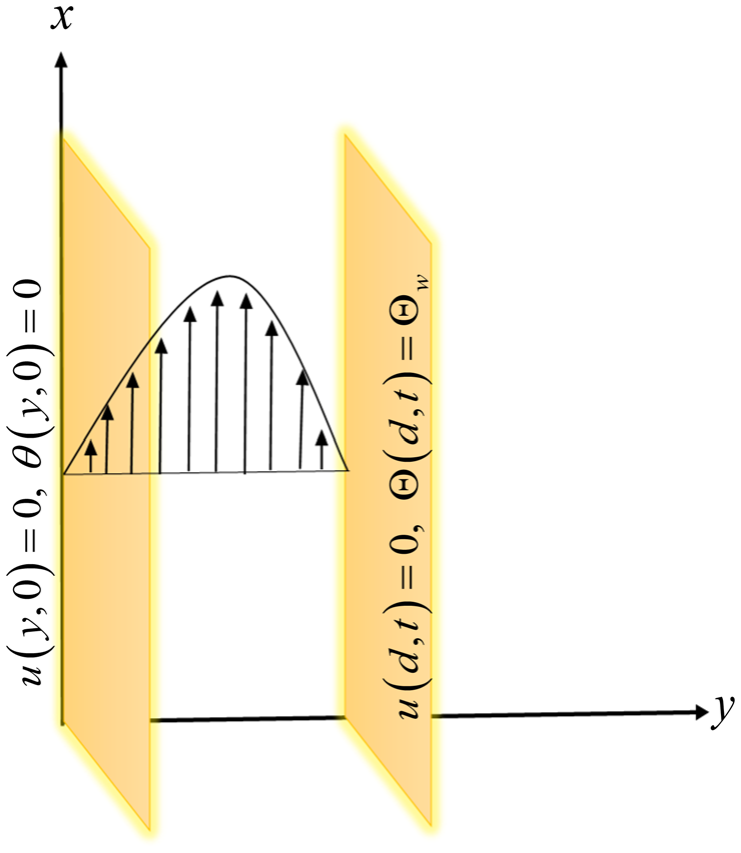
Schematic diagram of the flow.

Let us suppose an incompressible Newtonian viscous hybrid nanofluid between two infinite parallel plates. Water is taken as a based fluid with the combination of graphite and aluminium oxide nanoparticles. The distance between plates is $d$ in a coordinate system. It is assumed that both plates are at rest and the temperature of the left plate is $\Theta_{0}$, while the right plate temperature is $\Theta_{w}$. The flow of the fluid is due to free convection as shown in Fig. 2.

The Reynolds number is neglected, and no pressure gradient is assumed in the direction of flow. Maxwell’s relations for the magnetic field and Newton’s second law for velocity. Maxwell’s set of equations is as follow^33^:

1 $$ \left. {\begin{array}{*{20}l} {\nabla \cdot \overrightarrow {B} = 0,} \hfill \\ {\nabla \cdot \overrightarrow {E} = - \frac{{\partial \overrightarrow {B} }}{\partial t},} \hfill \\ {\nabla \cdot \overrightarrow {B} = \mu_{0} \overrightarrow {J} ,} \hfill \\ \end{array} } \right\} $$

where

2 $$ \overrightarrow {J} = \sigma_{0} \left[ {\overrightarrow {E} + \overrightarrow {V} \times \overrightarrow {B} } \right], $$

Moreover, the electromagnetic force is described as^33^

3 $$ \overrightarrow {{F_{em} }} = \overrightarrow {J} \times \overrightarrow {B} = \sigma_{0} \left[ {\overrightarrow {E} + \overrightarrow {V} \times \overrightarrow {B} } \right] \times \overrightarrow {B} = - \sigma B_{0}^{2} u\left( {y,t} \right){\text{i}}, $$

where ${\text{i}}$ is the unit vector in the x-direction. And $\overrightarrow {V} = \left( {u\left( {y,t} \right){\text{i}},0,0} \right)$ By incorporating $\overrightarrow {{F_{em} }}$ in momentum equation of unsteady incompressible we get the following set of partial differential equation^34^:

4 $$ \begin{aligned} \rho_{hnf} \frac{{\partial u\left( {y,t} \right)}}{\partial t} & = \mu_{hnf} \frac{{\partial^{2} u\left( {y,t} \right)}}{{\partial y^{2} }} + g\left( {\rho \beta_{\Theta } } \right)_{hnf} \left( {\Theta u\left( {y,t} \right) - \Theta_{0} } \right) - \sigma_{hnf} B_{0}^{2} u\left( {y,t} \right) \\ & \quad - \frac{{\mu_{hnf} }}{k}u\left( {y,t} \right). \\ \end{aligned} $$

where

$$ \mu_{hnf} = \frac{{\mu_{f} }}{{\left( {1 - \phi_{s1} } \right)^{2.5} \left( {1 - \phi_{s2} } \right)^{2.5} }}, $$

$$ \rho_{hnf} = \phi_{2} \rho_{s2} + \left( {1 - \phi_{s2} } \right)\left\{ {\left( {1 - \phi_{s1} } \right)\rho_{f} + \phi_{s1} \rho_{s1} } \right\}, $$

$$ \left( {\rho \beta_{\Theta } } \right)_{hnf} = \phi_{s2} \left( {\rho \beta_{\Theta } } \right)_{s2} + \left( {1 - \phi_{s2} } \right)\left\{ {\left( {1 - \phi_{s1} } \right)\left( {\rho \beta_{\Theta } } \right)_{f} + \phi_{s1} \left( {\rho \beta_{\Theta } } \right)_{s1} } \right\}. $$

The energy equation is

5 $$ \left( {\rho C_{p} } \right)_{hnf} \frac{{\partial \Theta \left( {y,t} \right)}}{\partial t} = K_{hnf} \frac{{\partial^{2} \Theta \left( {y,t} \right)}}{{\partial y^{2} }} + Q_{0} \left( {\Theta - \Theta_{0} } \right), $$

where $\sigma_{hnf}$, $\left( {\rho C_{p} } \right)_{hnf}$ and $K_{hnf}$ are the heat capacity and thermal conductivity of nanofluids defined as^35^:

$$ \begin{aligned} \sigma_{hnf} & = \sigma_{bf} \left\{ {\frac{{\sigma_{s2} \left( {1 + 2\phi_{s2} } \right) + 2\sigma_{bf} \left( {1 - \phi_{s2} } \right)}}{{\sigma_{s2} \left( {1 - \phi_{s2} } \right) + \sigma_{bf} \left( {2 + \phi_{s2} } \right)}}} \right\}, \\ \sigma_{bf} & = \sigma_{f} \left\{ {\frac{{\sigma_{s1} \left( {1 + 2\phi_{s1} } \right) + 2\sigma_{f} \left( {1 - \phi_{s1} } \right)}}{{\sigma_{s1} \left( {1 - \phi_{s1} } \right) + \sigma_{f} \left( {2 + \phi_{s1} } \right)}}} \right\} \\ \end{aligned} $$

$$ \begin{aligned} \left( {\rho C_{p} } \right)_{hnf} & = \phi_{s2} \left( {\rho C_{p} } \right)_{s2} + \left( {1 - \phi_{s2} } \right)\left\{ {\left( {1 - \phi_{s1} } \right)\left( {\rho C_{p} } \right)_{f} + \phi_{s1} \left( {\rho C_{p} } \right)_{s1} } \right\}, \\ \frac{{K_{bf} }}{{K_{f} }} & = \left\{ {\frac{{K_{s1} + \left( {m - 1} \right)K_{f} - \left( {m - 1} \right)\phi_{s1} \left( {K_{f} - K_{s1} } \right)}}{{K_{s1} + \left( {m - 1} \right)K_{f} + \phi_{s1} \left( {K_{f} - K_{s1} } \right)}}} \right\}, \\ \,\frac{{K_{hnf} }}{{K_{bf} }} & = \left\{ {\frac{{K_{s2} + \left( {m - 1} \right)K_{bf} - \left( {m - 1} \right)\phi_{s2} \left( {K_{bf} - K_{s2} } \right)}}{{K_{s2} + \left( {m - 1} \right)K_{bf} + \phi_{s2} \left( {K_{bf} - K_{s2} } \right)}}} \right\}\,. \\ \end{aligned} $$

The physical IBC are:

6 $$ u\left( {y,0} \right) = 0,\;\,\Theta \left( {y,0} \right) = \Theta_{0} \,\,{\text{for}}\;{\text{ all}}\, \, y \ge 0,\, $$

7 $$ u\left( {0,t} \right) = 0,\,\,\,\Theta \left( {0,t} \right) = \Theta_{0} ,\,\,t > 0,\, $$

8 $$ u\left( {d,t} \right) = 0,\,\,\,\Theta \left( {d,t} \right) = \Theta_{w} ,\,\,t > 0,\, $$

the subsequent dimensionless variables are introduced for non-dimensionalization:

9 $$ \;t^{*} = \frac{{\nu_{f} }}{{d^{2} }}t,\,\;\,\theta = \frac{{\Theta - \Theta_{0} }}{{\Theta_{w} - \Theta_{0} }},y^{*} = \frac{y}{d},\,\;u^{*} = \frac{d}{{\nu_{f} }}u,\, $$

used into Eqs. (2)–(8), we get (for simplicity dropped * sign)

10 $$ g_{1} \frac{\partial u}{{\partial t}}\left( {y,t} \right) = g_{2} \frac{{\partial^{2} u}}{{\partial y^{2} }}\left( {y,t} \right) + g_{3} Gr_{1} \theta \left( {y,t} \right) - g_{6} Mu\left( {y,t} \right) - \frac{1}{{k_{1} }}g_{2} u\left( {y,t} \right),\, $$

11 $$ g_{4} \Pr \frac{{\partial \theta \left( {y,t} \right)}}{\partial t} = Q\theta \left( {y,t} \right) + g_{5} \frac{{\partial^{2} \theta \left( {y,t} \right)}}{{\partial y^{2} }}. $$

12 $$ u\left( {y,0} \right) = 0,\;\,\theta \left( {y,0} \right) = 0\,\,{\text{for all}}\, \, y \ge 0,\, $$

13 $$ u\left( {0,t} \right) = 0,\,\,\,\theta \left( {0,t} \right) = 0,\,\,t > 0,\, $$

14 $$ u\left( {1,t} \right) = 0,\,\,\,\theta \left( {1,t} \right) = 1,\,\,t > 0,\, $$

where

$$ \Pr = \frac{{\mu_{f} \left( {C_{p} } \right)_{f} }}{{K_{f} }},g_{1} = \left( {1 - \phi_{s2} } \right)\left( {1 - \phi_{s1} + \phi_{s1} \frac{{\rho_{s1} }}{{\rho_{f} }}} \right) + \phi_{s2} \frac{{\rho_{s2} }}{{\rho_{f} }},Gr_{1} = \frac{{g\left( {\beta_{\Theta } } \right)_{f} \left( {\Theta_{w} - \Theta } \right)_{\infty } }}{{U^{3} }}, $$

$$ \begin{aligned} g_{2} & = \frac{1}{{\left( {1 - \phi_{s1} } \right)^{2.5} \left( {1 - \phi_{s2} } \right)^{2.5} }},g_{3} = \left( {1 - \phi_{s2} } \right)\left\{ {1 - \phi_{s1} + \phi_{s1} \frac{{\left( {\rho \beta_{\Theta } } \right)_{s1} }}{{\left( {\rho \beta_{\Theta } } \right)_{f} }}} \right\} + \phi_{s2} \frac{{\left( {\rho \beta_{\Theta } } \right)_{s2} }}{{\left( {\rho \beta_{\Theta } } \right)_{f} }},\,Q = \frac{{Q_{0} d^{2} }}{{K_{f} }} \\ g_{4} & = \left( {1 - \phi_{s2} } \right)\left\{ {1 - \phi_{s1} + \phi_{s1} \frac{{\left( {\rho C_{p} } \right)_{s1} }}{{\left( {\rho C_{p} } \right)_{f} }}} \right\} + \phi_{2} \frac{{\left( {\rho C_{p} } \right)_{s2} }}{{\left( {\rho C_{p} } \right)_{f} }},\,g_{5} = \left( {\frac{{K_{hnf} }}{{K_{f} }}} \right),\,g_{6} = \frac{{\sigma_{hnf} }}{{\sigma_{bf} }},k_{1} = \frac{k}{{d^{2} }} \\ \frac{{K_{hnf} }}{{K_{f} }} & = \left\{ {\frac{{K_{s1} + \left( {m - 1} \right)K_{f} - \left( {m - 1} \right)\phi_{s1} \left( {K_{f} - K_{s1} } \right)}}{{K_{s1} + \left( {m - 1} \right)K_{f} + \phi_{s1} \left( {K_{f} - K_{s1} } \right)}}} \right\}\left\{ {\frac{{K_{s2} + \left( {m - 1} \right)K_{bf} - \left( {m - 1} \right)\phi_{s2} \left( {K_{bf} - K_{s2} } \right)}}{{K_{s2} + \left( {m - 1} \right)K_{bf} + \phi_{s2} \left( {K_{bf} - K_{s2} } \right)}}} \right\}. \\ \end{aligned} $$

## Solution with fractional model

**Figure 3:**
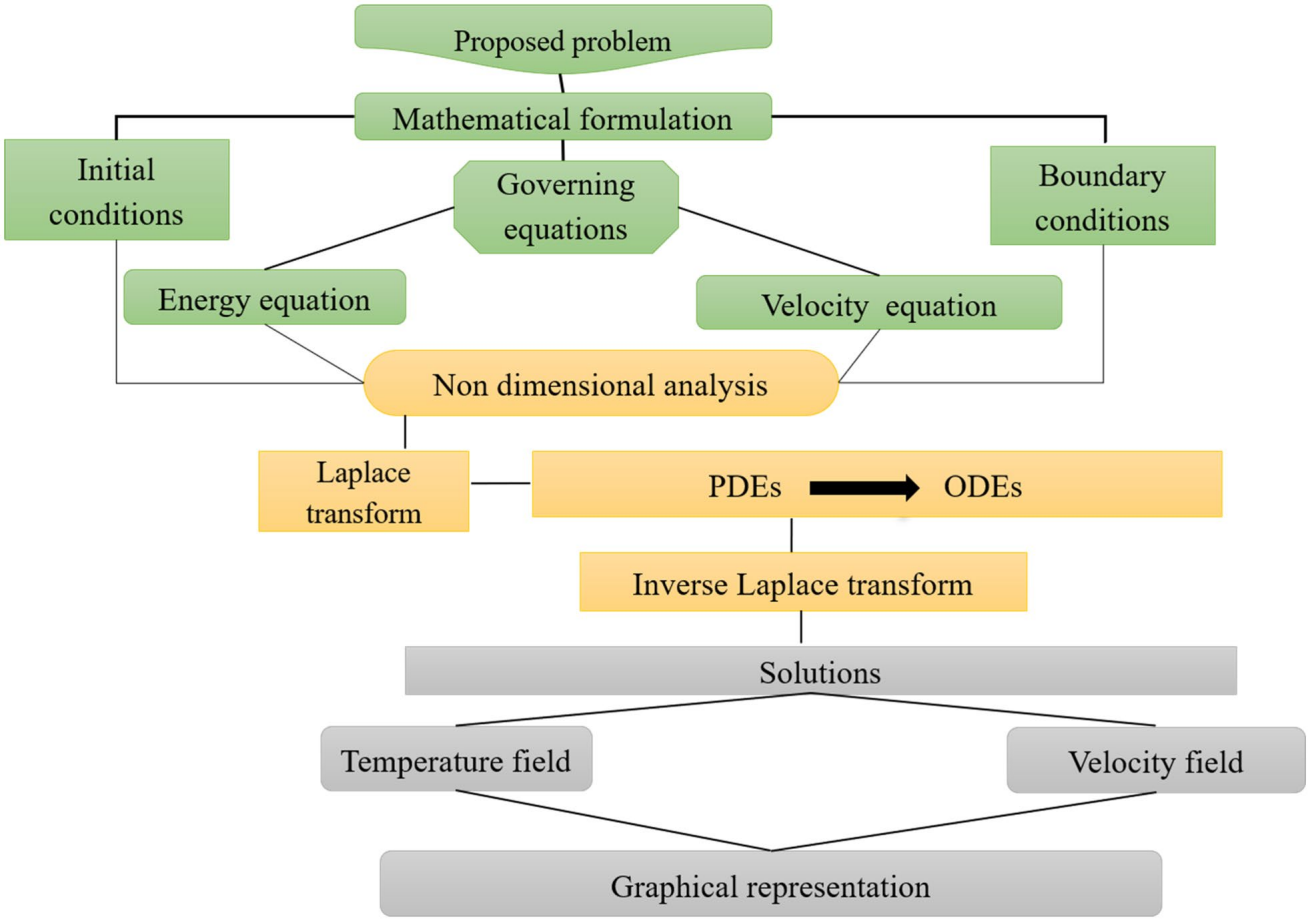
Flow chart of the steps-wise procedure.

The flow chart of the problem is summarized in Fig. 3. Furthermore, the Caputo time fractional model of Eqs. (10) and (11) as follows:

15 $$ g_{1} {}^{C}D_{t}^{\alpha } u\left( {y,t} \right) = g_{2} \frac{{\partial^{2} u\left( {y,t} \right)}}{{\partial y^{2} }} + g_{3} Gr_{1} \theta \left( {y,t} \right) - g_{6} Mu\left( {y,t} \right) - \frac{1}{{k_{1} }}g_{2} u\left( {y,t} \right), $$

16 $$ g_{4} \Pr {}^{C}D_{t}^{\alpha } \theta \left( {y,t} \right) = g_{5} \frac{{\partial^{2} \theta \left( {y,t} \right)}}{{\partial y^{2} }} + Q\theta \left( {y,t} \right). $$

where

$$ {}^{C}D_{t}^{\alpha } f\left( {y,t} \right) = \left\{ {\begin{array}{*{20}l} {\frac{1}{{\Gamma \left( {1 - \alpha } \right)}}\int\limits_{0}^{t} {\frac{1}{{\left( {t - \tau } \right)^{\alpha } }}\frac{{\partial f\left( {y,\tau } \right)}}{\partial \tau }d\tau ,} } \hfill & {0 < \alpha < 1} \hfill \\ {\frac{{\partial f\left( {y,\tau } \right)}}{\partial \tau },} \hfill & {\alpha = 1.} \hfill \\ \end{array} } \right. $$

Apply the Laplace transform of Eqs. (15) and (16) and simplify we get:

17 $$ \overline{\theta }\left( {y,q} \right) = \frac{{\sinh \left( {y\sqrt {\frac{{g_{9} q}}{{q + g_{8} }} + \frac{Q}{{g_{5} }}} } \right)}}{{\sinh \left( {\sqrt {\frac{{g_{9} q}}{{q + g_{8} }} + \frac{Q}{{g_{5} }}} } \right)}}\frac{1}{q} $$

18 $$ \overline{u}\left( {y,q} \right) = \frac{1}{q}\frac{{\frac{{g_{3} }}{{g_{2} }}Gr_{1} }}{{\frac{{g_{9} q}}{{q + g_{8} }} + \frac{Q}{{g_{5} }} - \sqrt {\frac{{\frac{{g_{3} }}{{g_{2} }}g_{1} q}}{{q + g_{8} }} + \frac{{g_{10} }}{{g_{2} }}} }}\left[ {\frac{{\sinh \left( {y\sqrt {\frac{{\frac{{g_{3} }}{{g_{2} }}g_{1} q}}{{q + g_{8} }} + \frac{{g_{10} }}{{g_{2} }}} } \right)}}{{\sinh \left( {\sqrt {\frac{{\frac{{g_{3} }}{{g_{2} }}g_{1} q}}{{q + g_{8} }} + \frac{{g_{10} }}{{g_{2} }}} } \right)}} - \frac{{\sinh \left( {y\sqrt {\frac{{g_{9} q}}{{q + g_{8} }} + \frac{Q}{{g_{5} }}} } \right)}}{{\sinh \left( {\sqrt {\frac{{g_{9} q}}{{q + g_{8} }} + \frac{Q}{{g_{5} }}} } \right)}}} \right], $$

Equations (17) and (18) present the solutions of Eqs. (15) and (16) in the transformed variable *q*. To obtain the inverse Laplace transform by using the inversion method by^36–39^, we get

19 $$ \theta \left( {y,t} \right) = \frac{2}{t}\sum\limits_{j = 1}^{N} {{\text{Re}} \left\{ {K_{j} \overline{\theta }\left( {y,\frac{{\alpha_{j} }}{t}} \right)} \right\}} $$

20 $$ u\left( {y,t} \right) = \frac{2}{t}\sum\limits_{j = 1}^{N} {{\text{Re}} \left\{ {K_{j} \overline{u}\left( {y,\frac{{\alpha_{j} }}{t}} \right)} \right\}} $$

where $K_{j}$ and $\alpha_{j}$ are given as;

**Table:** 

$j$	$K_{j}$	$\alpha_{j}$
1	12.83767675 + *i* 1.666063445	− 36,902.0821 + *i* 196,990.4257
2	12.22613209 + *i* 5.012718792	61,277.02524 − *i* 95,408.62551
3	10.93430308 + *i* 8.409673116	− 28,916.56288 + *i* 18,169.18531
4	8.776434715 + *i* 11.92185389	4655.361138 − *i* 1.901528642
5	5.225453361 + *i* 15.72952905	− 118.7414011 − *i* 141.3036911

## Graphical results and discussion

See Figs. 3, 4, 5, 6, 7, 8, 9, 10, 11 and 12.

**Figure 4:**
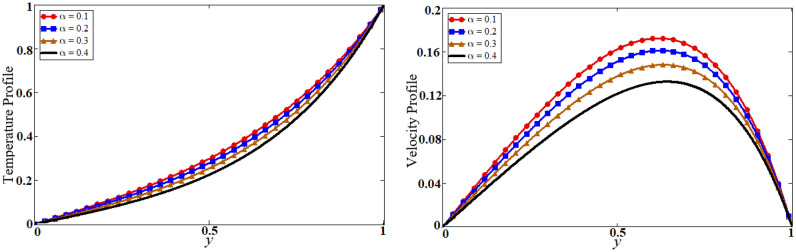
Results of the fractional parameter $\alpha$ on temperature and velocity profile.

**Figure 5:**
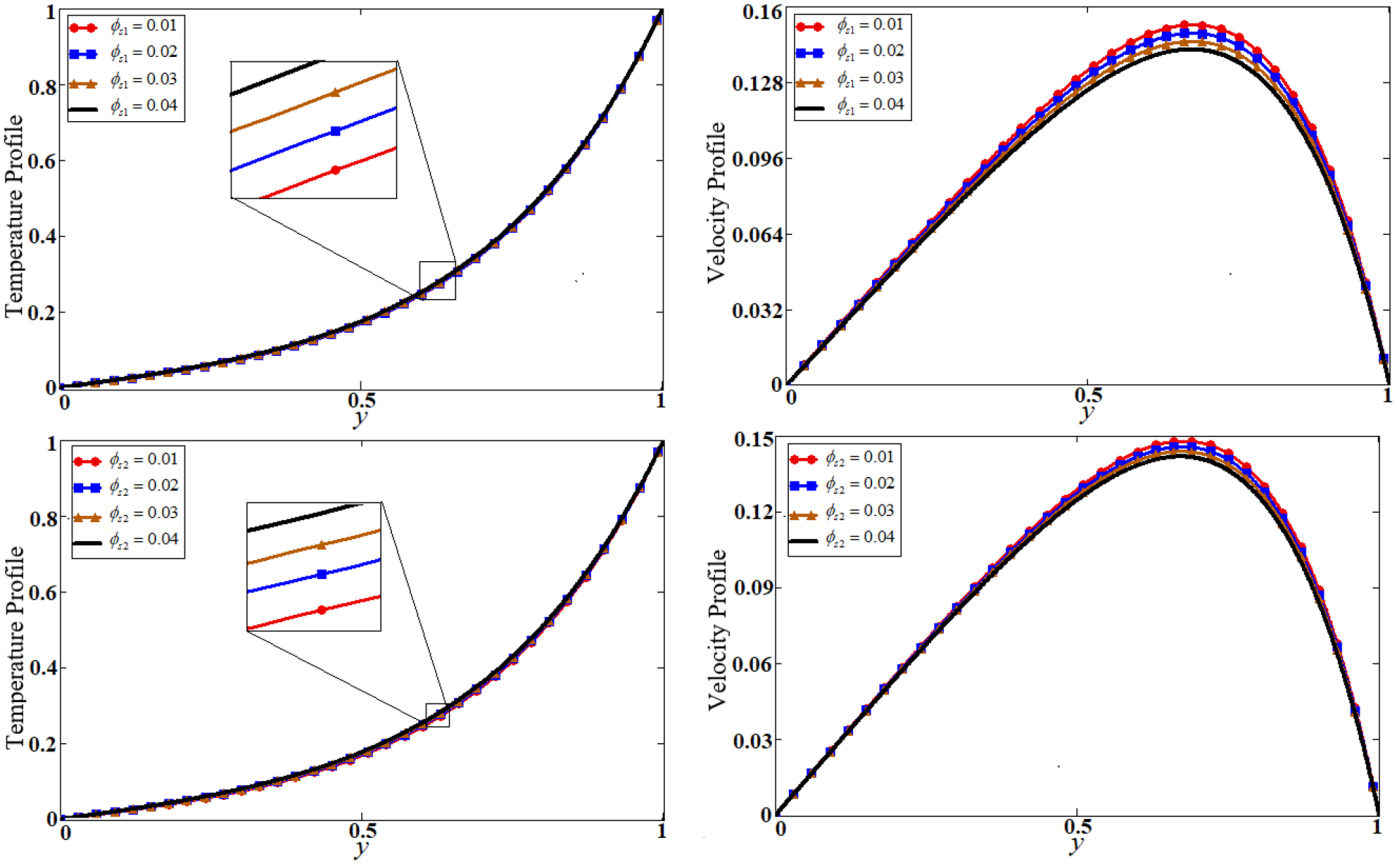
Results of volume fractional of both nanoparticles on temperature and velocity.

**Figure 6:**
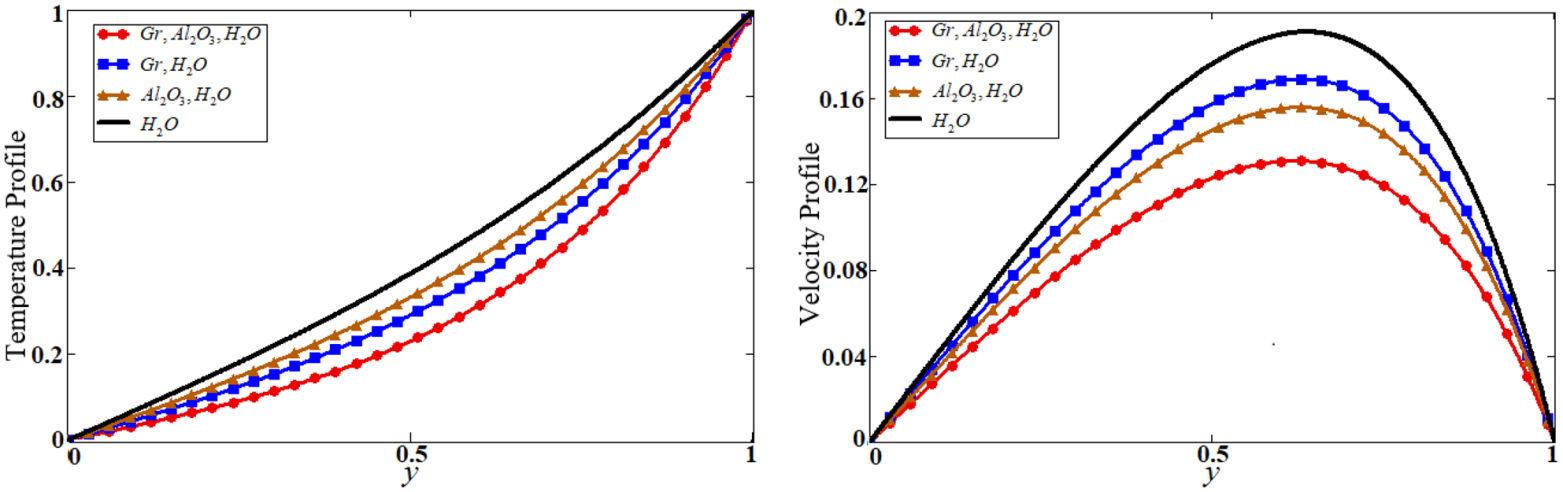
Comparison of hybrid nanofluid with nanofluid on temperature and velocity profile.

**Figure 7:**
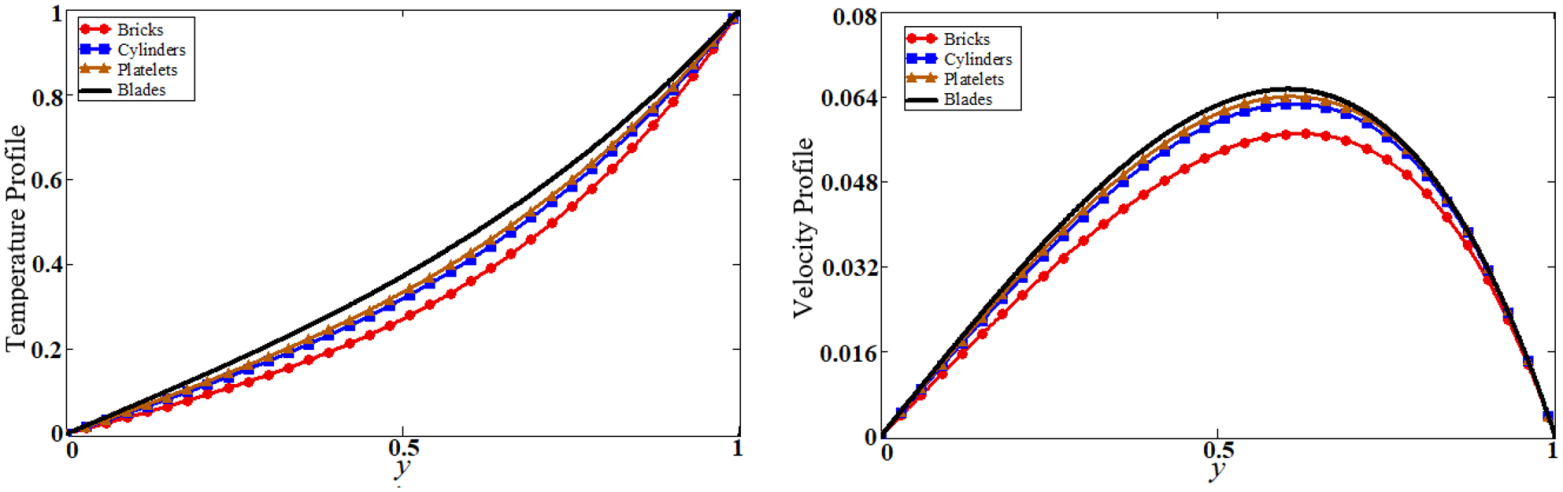
The shape effect of nanoparticles on temperature and velocity.

**Figure 8:**
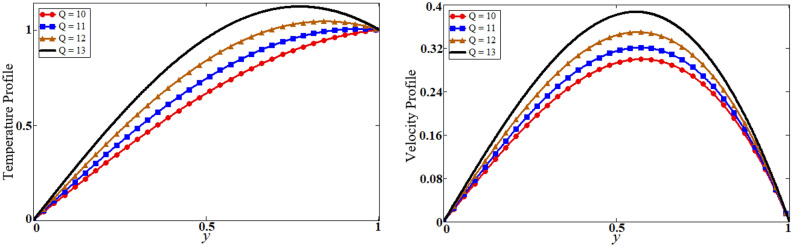
Results of heat generation $Q$ on temperature and velocity.

**Figure 9:**
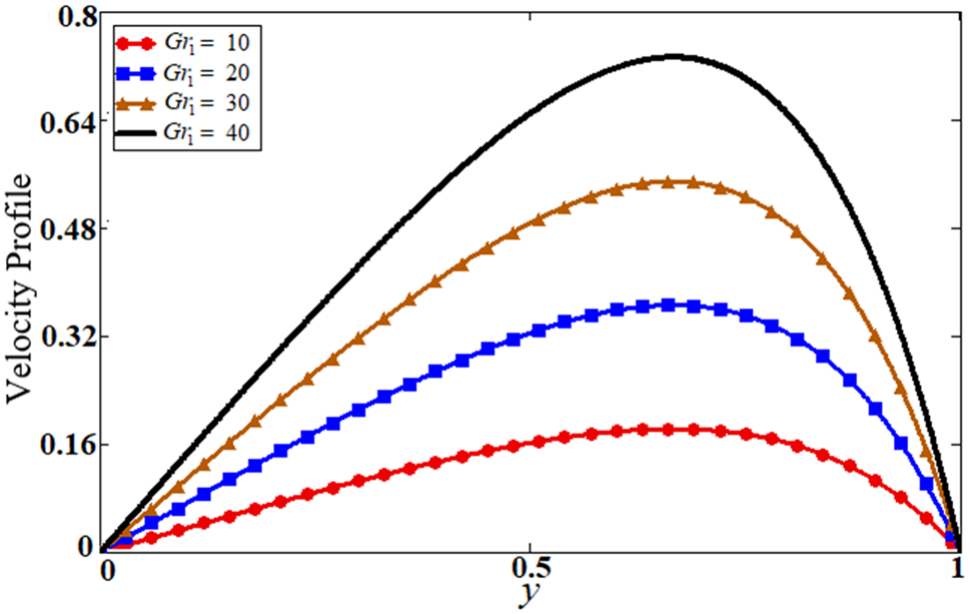
Results of $Gr_{1}$ on velocity.

**Figure 10:**
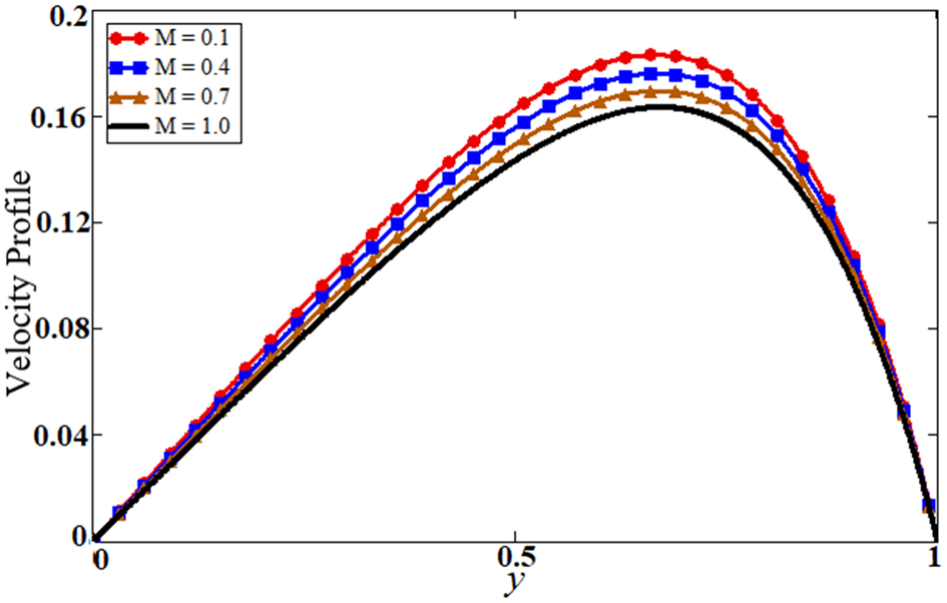
Results of magnetic parameter M on velocity.

**Figure 11:**
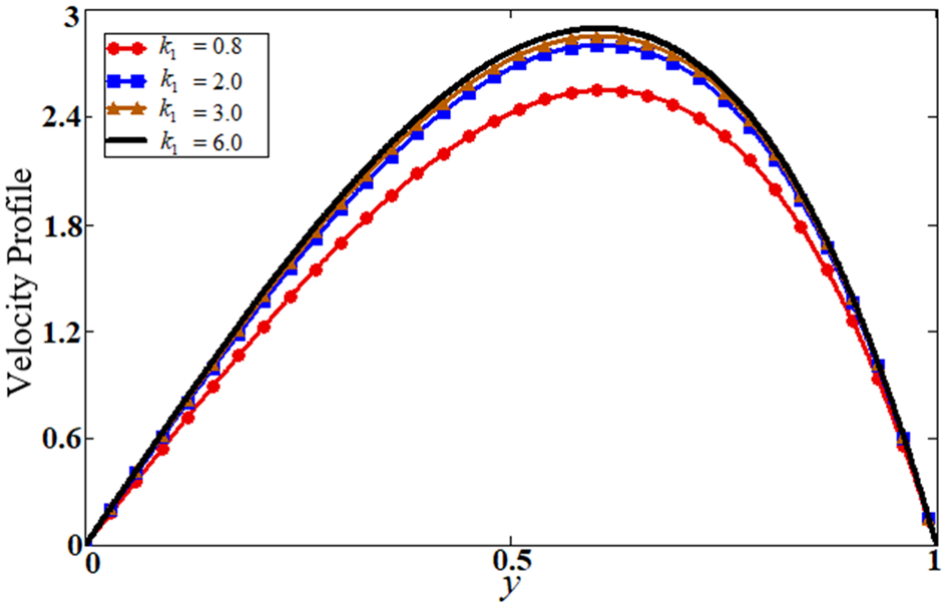
Results of porosity parameter $k_{1}$ on velocity.

**Figure 12:**
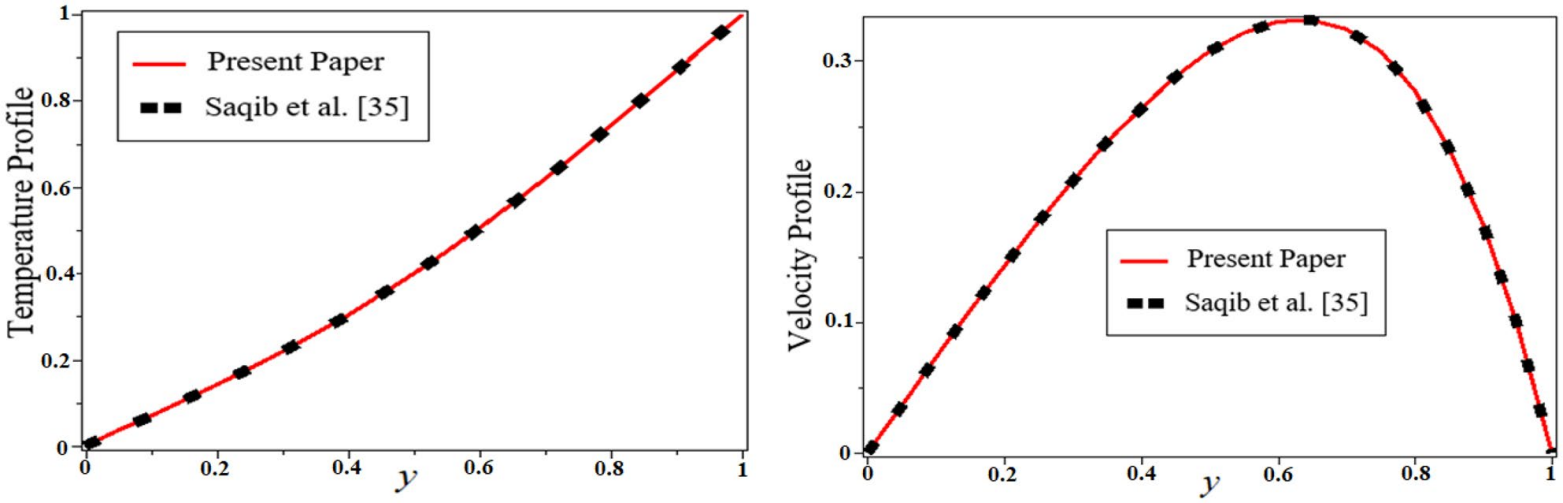
Comparison plot of the present solution with Saqib et al*.*^40^.

## Results and discussion

The problem of hybrid nanofluid is solved by using the Laplace transform method. Results are computed and discussed for several embedded parameters. Results are based on the nanoparticle's thermophysical properties in Table 1. Results plotted in Fig. 4 reflects the effect of fractional parameter $\alpha$ on velocity and temperature distributions. Different graphs for different values of $\alpha$ obtained for velocity and temperature profiles. These graphs are obtained for various values of $\alpha$ while keeping other parameters constants. In addition, each graph is made for a distinct value of $\alpha$, which represents a solution so in this manner we have obtained various solutions. For the experimentalists, it is easy to select the best curve fitting graph, which can be in good agreement with the results of real data. When $\phi$ is increased from 0.01 to 0.04, the velocity of the hybrid nanofluid is decreased for both particles, $\phi_{s1}$ for one particle and $\phi_{s2}$ for the second one. Physically this phenomenon says that with increasing values of volume fraction, the viscous forces become stronger due to which velocity retards. This physical trend is noted in Fig. 5 for both nanoparticles volume fractions. Which makes a strong agreement with the physics of the volume fraction of hybrid nanofluids. The comparison of $Al_{2} O_{3} ,\,Gr$ (hybrid nanofluid) is shown with respect to water-base $Gr$ and $Al_{2} O_{3}$ nanofluid in Fig. 6. It is observed that the velocity of the $Al_{2} O_{3} ,\,Gr$ (hybrid nanofluid) is lower as compared to other nanofluids. Physically, this is true because the density of $Al_{2} O_{3} ,\,Gr$ (hybrid nanofluid) is greater than the density of other nanofluids which is a factor to decrease the velocity. Moreover, the temperature distribution shows the same behavior as that of the velocity profile. As $Al_{2} O_{3} ,\,Gr$ (hybrid nanofluid) has higher thermal conductivity than other nanofluids but less density. Consequently, $Al_{2} O_{3} ,\,Gr$ (hybrid nanofluid) transfers more heat compared to other nanofluids and are less dense, as a result, the temperature distributions of $Al_{2} O_{3} ,\,Gr$ (hybrid nanofluid) is lower than other nanofluids. The effect of the shape factor on the velocity and temperature profiles has been depicted in Fig. 7. The hydrogen bonding of hybrid nanofluid produces an important argument in thermal conductivity thus the velocity and temperature distribution are enhanced. The temperature profile for blade shape hybrid nanoparticles is maximum while for brick shape nanoparticles the temperature distribution is minimum. Similarly, the velocity profile for blade shape is maximum and minimum for brick shape nanoparticles. Figure 8 illustrates the influence of heat generation in temperature and velocity field, it is notified that heat generation boosts the temperature and velocity. When $Q$ is set to a higher value, it indicates that the device has absorbed more heat, resulting in a weaker intermolecular attractive force, which causes the temperature and velocity profiles to rise. Figure 9 reflects the influence of $Gr_{1}$ on the velocity of hybrid nanofluid. $Gr_{1}$ is the ratio of thermal to viscous forces, the dominancy of thermal forces for the fluid velocity for enhancement? That is why the increasing behavior of velocity of hybrid nanofluid is pictured in Fig. 9. Figure 10 stipulates that enhancing the magnetic parameter $M$ provides a considerable hindrance to the flow behavior. This hindrance is due to the fact that enlargement of $M$ strengthens the Lorentz forces, which by nature are resistive forces. Therefore, due to these strong resistive forces’ retardation occurs in the fluid flow, and a decrease in boundary layer thickness is seen.

**Table 1:** Base fluid and nanoparticles thermophysical properties^9^.

Material	Base fluids	Nanoparticles	
	Water	${\text{Al}}_{2} {\text{O}}_{3}$	${\text{Gr}}$
$\rho \left( {{{{\text{kg}}} \mathord{\left/ {\vphantom {{{\text{kg}}} {{\text{m}}^{{3}} }}} \right. \kern-\nulldelimiterspace} {{\text{m}}^{{3}} }}} \right)$	997.1	3970	1300
$c_{p} \left( {{{\text{J}} \mathord{\left/ {\vphantom {{\text{J}} {{\text{kg}}\,{\text{K}}}}} \right. \kern-\nulldelimiterspace} {{\text{kg}}\,{\text{K}}}}} \right)$	4179	765	830
$K\left( {{{\text{W}} \mathord{\left/ {\vphantom {{\text{W}} {{\text{m}}\,{\text{K}}}}} \right. \kern-\nulldelimiterspace} {{\text{m}}\,{\text{K}}}}} \right)$	0.613	40	12
$\beta \times 10^{ - 5} \left( {{\text{K}}^{{ - 1}} } \right)$	21	0.85	2.83
$\sigma \left( {\Omega {\text{m}}} \right)$	0.05	3.5*10^6	3.3
$\Pr$	6.2	–	–

The last Fig. 11 illustrates the effect of the porosity parameter $k_{1}$ on velocity profile, which enhances the velocity of the fluid. This is due to a decrease in the resistance of the porous medium, which leads to an increase in the thickness of the momentum boundary layer. By putting $\alpha = 0.5,\,M = 1.5,\,\,Gr = 5,\,\,k_{1} = 15,\,t = 1,Q = 0$ and $\,\phi_{s1} = \phi_{s2} = 0$ our solution is reduced to the solution of Saqib et al.^40^, which is presented in Fig. 12. Table 2 highlights the variation of Nusselt number for classical, unitary, and hybrid nanofluids nanoparticles, comparative analysis with nanofluid and shape effect of nanoparticles. It is observed that the enhancement of the rate of heat transfer is boosting up to 37.40741% by using hybrid nanofluid compared to unitary nanofluids, and up to 11.14983% by adding differently shaped nanoparticles in the base fluids. The variation of skin fraction for both plates is present in Table 3. It is observed from this table that increasing the volume fraction of nanofluid increases the skin faction on a left plate while the opposite behavior on the right plate. It is also clear that blades shaped nanoparticles have a high skin fraction on a left plate while Brick’s shape nanoparticles have a high skin fraction on the right plate.

**Table 2:** The percentage variation of Nusselt number.

$\phi_{s1} + \phi_{s2}$	Nu	%age
0	0.27	
0.01	0.294	8.888889
0.02	0.319	18.14815
0.03	0.344	27.40741
0.04	0.371	37.40741

Different fluids	Nu	%age
Water	0.27	0
*Gr*	0.313	15.92593
*Al*_*2*_*O*_*3*_	0.321	18.88889
*Gr-Al*_*2*_*O*_*3*_	0.371	37.40741

Shape, m	Nu	%age
Bricks, 3.9	0.287	0
Cylinders, 4.9	0.296	3.135889
Platelets, 5.7	0.303	5.574913
Blades, 8.6	0.319	11.14983

**Table 3:** The variation of skin fraction on both plates.

$\phi_{s1} + \phi_{s2}$	$Cf$ at $y = 0$	$Cf$ at $y = 1$
0	0.635	− 1.692
0.01	0.667	− 1.743
0.02	0.719	− 1.841
0.03	0.795	− 2
0.04	0.887	− 2.196

Different fluids	$Cf$ at $y = 0$	$Cf$ at $y = 1$
Water	0.635	− 1.692
*Gr*	0.66	− 1.737
*Al*_*2*_*O*_*3*_	0.638	− 1.689
*Gr-Al*_*2*_*O*_*3*_	0.667	− 1.743

Shape, m	$Cf$ at $y = 0$	$Cf$ at $y = 1$
Bricks, 3.9	0.667	− 1.743
Cylinders, 4.9	0.674	− 1.753
Platelets, 5.7	0.679	− 1.76
Blades, 8.6	0.7	− 1.796

## Summery

In the water filtration process application, this mathematical model is developed and analyzed with the combination of nanoparticles which make hybrid nanofluid. The results (graphical and tabulated) are purely obtained by numerical inversion of the Laplace transform. The different parameters are analyzed for the velocity and temperature profile. The comparison is also explored for nanofluid and hybrid nanofluid with graphite and aluminium oxide nanoparticles. The main outcomes are as follows.

- The fractional parameter provides the range of solution for temperature and velocity profile which makes the analysis more general as compared to the classical one.
- The hybrid nanofluid has enhanced the temperature and retard the velocity, which helps in the rate of heat transfer during the drilling process.
- The shape effect of nanoparticles reduced the temperature and velocity profile, which means that different shapes of nanoparticles should be used to control the temperature and velocity of the hybrid nanofluid during the filtration process by adding different shapes of the nanoparticle.
- Dissolving the hybrid nanocomposite ($Al_{2} O_{3} ,\,Gr$) in a water-base enhances the rate of heat transfer up to 37.40741%, which is higher than unitary nanofluid.
- The blade's shape of nanoparticles enhances the rate of heat transfer up to 11.14983% rather than other shapes of nanoparticles during the filtration process.
- The skin fraction on both plates can be controlled by adding hybrid nanofluid, the volume fraction of nanoparticles, and by adding different shapes of nanoparticles.

Lastly, I endorse some suggestions for future research to the readers as below.

- The following concept can be applied to various geometries of calendrical coordinates.
- One way to explore the same concept for different types of fluids with slip boundary conditions.
- Furthermore, some new fractional derivatives can be used to more effectively summarise present fluid models along with concertation equations.

## Data Availability

Data of this study will be made available from the corresponding author on reasonable request.

## List of symbols

$C_{p}$: Heat capacity at a constant pressure, ${\text{kg}}^{ - 1} \;{\text{K}}^{ - 1}$
$k_{1}$: Dimensionless porosity parameter
$Gr$: Thermal Grashof number
$g$: Acceleration due to gravity, ${\text{m}}\;{\text{s}}^{ - 2}$
$K$: Base fluid thermal conductivity, ${\text{W}}\;{\text{m}}^{ - 1} \;{\text{K}}^{ - 1}$
$B_{0}$: Applied magnetic field, ${\text{T}}$
$\sigma$: Electrical conductivity, $\left( {\frac{{\text{S}}}{{\text{m}}}} \right)$ S is Siemens
$$Pr$$: Prandtl number
$$t$$: Time, s
$u$: Velocity of the fluid, ${\text{m}}\;{\text{s}}^{ - 1}$
$$Q$$: Heat generation
$$M$$: Magnetic parameter
$$Al_{2} O_{3}$$: Aluminium oxide
$\vec{B}$: Magnetic flux intensity, ${\text{A}}\,{\text{m}}^{ - 1}$
$$\mu_{0}$$: Magnetic permeability, $${\text{N}}\,{\text{A}}^{ - 1}$$
$$\sigma_{0}$$: Electrical conductivity, $${\text{S}}\,{\text{m}}^{ - 1}$$
$$i$$: Unit vector
$\beta_{\Theta }$: Volumetric coefficient of thermal expansion, ${\text{K}}^{ - 1}$
$$\theta$$: Dimensionless temperature
$\Theta$: Temperature of the fluid, K
$$\Theta_{\infty }$$: Ambient temperature, K
$\Theta_{w}$: Wall temperature K is used as a unit as well as thermal conductivity, K
$$\left[ \cdot \right]_{f}$$: Base fluid
$$\left[ \cdot \right]_{nf}$$: Nanofluid
$$\left[ \cdot \right]_{s1,s2}$$: Solid particles
$$\rho$$: Density, $${\text{kg}}\;{\text{m}}^{ - 3}$$
$\phi$: Nanoparticle volume fraction
$$\mu$$: Dynamic viscosity, $${\text{kg}}\;{\text{m}}^{ - 1} \;{\text{s}}^{ - 1}$$
$$Gr$$: Graphite
$d$: Distance between two plates, m
$\vec{E}$: Electric field intensity, ${\text{V}}\,{\text{m}}^{ - 1}$
$$\vec{J}$$: Current density, $${\text{A}}\,{\text{m}}^{ - 2}$$
$$\vec{V}$$: Velocity vector
